# Residual macrovascular risk in 2013: what have we learned?

**DOI:** 10.1186/1475-2840-13-26

**Published:** 2014-01-24

**Authors:** Jean-Charles Fruchart, Jean Davignon, Michel P Hermans, Khalid Al-Rubeaan, Pierre Amarenco, Gerd Assmann, Philip Barter, John Betteridge, Eric Bruckert, Ada Cuevas, Michel Farnier, Ele Ferrannini, Paola Fioretto, Jacques Genest, Henry N Ginsberg, Antonio M Gotto, Dayi Hu, Takashi Kadowaki, Tatsuhiko Kodama, Michel Krempf, Yuji Matsuzawa, Jesús Millán Núñez-Cortés, Carlos Calvo Monfil, Hisao Ogawa, Jorge Plutzky, Daniel J Rader, Shaukat Sadikot, Raul D Santos, Evgeny Shlyakhto, Piyamitr Sritara, Rody Sy, Alan Tall, Chee Eng Tan, Lale Tokgözoğlu, Peter P Toth, Paul Valensi, Christoph Wanner, Alberto Zambon, Junren Zhu, Paul Zimmet

**Affiliations:** 1 R3i Foundation, St. Alban-Anlage 46, Basel, CH 4010, Switzerland; 2 Fondation Cœur et Artères, Lille, France; 3 Institut de recherches cliniques de Montréal; Centre Hospitalier de l’Université de Montréal and Department of Experimental Medicine, McGill University, Montreal, Canada; 4 Cliniques Universitaires Saint-Luc, Brussels, Belgium; 5 University Diabetes Center, King Saud University, Riyadh, Saudi Arabia; 6 Department of Neurology and Stroke Centre, Bichat University Hospital, Paris, France; 7 Assmann-Stiftung für Prävention, Münster, Germany; 8 Centre for Vascular Research, University of New South Wales, Sydney, Australia; 9 University College London, London, UK; 10 Department of Endocrinology and Cardiovascular Disease Prevention, Institut of CardioMetabolism and Nutrition (ICAN) Hôpital Pitié-Salpêtrière, Paris, France; 11 Nutrition Center, Clínica Las Condes, Santiago, Chile; 12 Point Medical, Dijon, France; 13 University of Pisa School of Medicine, and Metabolism Unit of the National Research Council (CNR) Institute of Clinical Physiology, Pisa, Italy; 14 Department of Medical and Surgical Sciences, University of Padova, Padova, Italy; 15 McGill University and Center for Innovative Medicine, McGill University Health Center/Royal Victoria Hospital, Montreal, Canada; 16 Department of Medicine and Irving Institute for Clinical and Translational Research, Columbia University, New York, USA; 17 Weill Cornell Medical College, Cornell University, New York, USA; 18 Heart Institute, People Hospital of Peking University, Beijing, China; 19 Department of Diabetes and Metabolic Diseases Unit, The University of Tokyo, Tokyo, Japan; 20 Department of Systems Biology and Medicine, The University of Tokyo, Tokyo, Japan; 21 Human Nutritional Research Center and Department of Endocrinology, Metabolic Diseases and Nutrition, University Hospital Nantes, Nantes, France; 22 Sumitomo Hospital and Osaka University, Osaka, Japan; 23 University Hospital Gregorio Marañón, Universidad Complutense, Madrid, Spain; 24 University of Concepción, Concepción, Chile; 25 Department of Cardiovascular Medicine, Kumamoto University, Kumamoto, Japan; 26 Brigham and Women’s Hospital and Harvard Medical School, Boston, USA; 27 Division of Translational Medicine and Human Genetics, Smilow Center for Translational Research, Penn Cardiovascular Institute, Philadelphia, PA, USA; 28 Jaslok Hospital and Research Center, Mumbai, India; 29 Unidade Clínica de Lipides InCor-HCFMUSP, Sao Paulo, Brazil; 30 Federal Almazov Heart Blood Endocrinology Centre, St Petersburg, Russia; 31 Mahidol University, Bangkok, Thailand; 32 University of the Philippines-Philippine General Hospital, Manila, The Philippines; 33 Specialized Center of Research (SCOR) in Molecular Medicine and Atherosclerosis, Columbia University, College of Physicians & Surgeons, New York, USA; 34 Gleneagles Medical Centre, Singapore; 35 Hacettepe University, Ankara, Turkey; 36 Sterling Rock Falls Clinic, CGH Medical Center, Sterling and University of Illinois School of Medicine, Peoria, IL, USA; 37 Hôpital Jean Verdier, Department of Endocrinology Diabetology Nutrition, AP-HP, Paris-Nord University, CRNH-IdF, CINFO, Bondy, France; 38 University Hospital Würzburg, Würzburg, Germany; 39 Zhongshan Hospital, Fudan University, Shanghai, China; 40 Baker IDI Heart and Diabetes Institute, Melbourne, Australia

**Keywords:** Residual cardiovascular risk, Atherogenic dyslipidaemia, Type 2 diabetes, Therapeutic options

## Abstract

Cardiovascular disease poses a major challenge for the 21st century, exacerbated by the pandemics of obesity, metabolic syndrome and type 2 diabetes. While best standards of care, including high-dose statins, can ameliorate the risk of vascular complications, patients remain at high risk of cardiovascular events. The Residual Risk Reduction Initiative (R^3^i) has previously highlighted atherogenic dyslipidaemia, defined as the imbalance between proatherogenic triglyceride-rich apolipoprotein B-containing-lipoproteins and antiatherogenic apolipoprotein A-I-lipoproteins (as in high-density lipoprotein, HDL), as an important modifiable contributor to lipid-related residual cardiovascular risk, especially in insulin-resistant conditions. As part of its mission to improve awareness and clinical management of atherogenic dyslipidaemia, the R^3^i has identified three key priorities for action: i) to improve recognition of atherogenic dyslipidaemia in patients at high cardiometabolic risk with or without diabetes; ii) to improve implementation and adherence to guideline-based therapies; and iii) to improve therapeutic strategies for managing atherogenic dyslipidaemia. The R^3^i believes that monitoring of non-HDL cholesterol provides a simple, practical tool for treatment decisions regarding the management of lipid-related residual cardiovascular risk. Addition of a fibrate, niacin (North and South America), omega-3 fatty acids or ezetimibe are all options for combination with a statin to further reduce non-HDL cholesterol, although lacking in hard evidence for cardiovascular outcome benefits. Several emerging treatments may offer promise. These include the next generation peroxisome proliferator-activated receptorα agonists, cholesteryl ester transfer protein inhibitors and monoclonal antibody therapy targeting proprotein convertase subtilisin/kexin type 9. However, long-term outcomes and safety data are clearly needed. In conclusion, the R^3^i believes that ongoing trials with these novel treatments may help to define the optimal management of atherogenic dyslipidaemia to reduce the clinical and socioeconomic burden of residual cardiovascular risk.

## Introduction

Cardiovascular disease (CVD) remains the leading cause of death and a major cause of disability affecting quality of life [1,2]. Despite best evidence-based strategies, including high-dose statin therapy, it is clear that there persists an unacceptably high residual risk of CV events. According to the Residual Risk Reduction Initiative (R^3^i), residual CV risk is defined as the risk of CV events that persists in people despite achievement of treatment goals for low-density lipoprotein (LDL) cholesterol, blood pressure, and glycaemia according to current standards of care.

**Figure 1:**
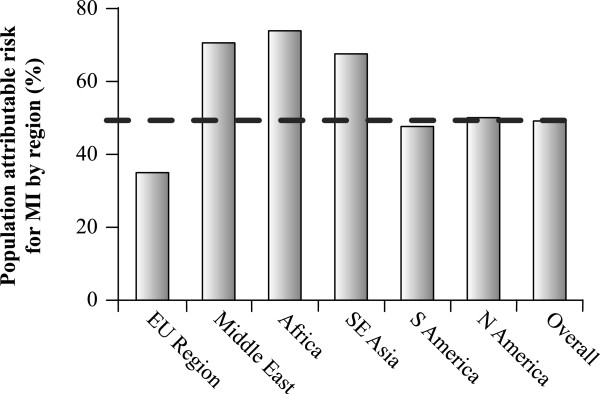
**Population**-**attributable coronary risk due to dyslipidaemia across different regions in the INTERHEART study **[5]**.** Dyslipidaemia was defined as the ratio of apolipoprotein B-containing lipoproteins to apolipoprotein A-I lipoproteins.

This clinical challenge is exacerbated by the pandemics of obesity, metabolic syndrome and type 2 diabetes. Diabetes prevalence is increasing in almost every country; globally, it is estimated that diabetes affects over 371 million people and costs US$471 billion [3]. However, the burden of diabetes is likely to be even greater in emerging economies in Asia, Africa and the Middle East. The reasons for this are multifactorial and include transition to an increasingly urbanised, sedentary society resulting in increasing obesity across the socioeconomic spectrum, as well as early life influences, such as maternal nutrition and newborn overfeeding, which are associated with epigenetic changes that increase the risk of obesity, diabetes and CVD in later life [4]. Thus, individuals in these regions have increased susceptibility to cardiometabolic abnormalities at lower absolute levels of adiposity. Indeed, this scenario is illustrated by the INTERHEART study, a global case–control study which highlighted the relevance of both atherogenic apolipoprotein (apo) B100-containing lipoproteins and potentially atheroprotective apoA-I containing lipoproteins, such as high-density lipoproteins (HDL), to coronary risk. The population-attributable coronary risk due to dyslipidaemia (defined as the ratio apoB/apoA-I) was almost double in some of these emerging economic regions compared with the European Union region (Figure 1) [5]. In Latin America, abdominal obesity, dyslipidaemia and smoking collectively accounted for 88% of the population-attributable coronary risk [6].

The Residual Risk Reduction Initiative (R^3^i) believes that residual vascular risk represents a paramount public health challenge in the 21st century [7]. Thus, the mission of this worldwide, academic initiative is to raise awareness of the numerous factors influencing residual vascular risk, with a focus on lipoprotein-related risk factors, and to improve strategies for its therapeutic management. Five years ago, the R^3^i identified atherogenic dyslipidaemia, defined as the imbalance between proatherogenic apoB-containing lipoproteins (contained in triglyceride-rich lipoproteins, TRLs) and antiatherogenic apo A-I-lipoproteins (contained in HDL), as a key contributor to residual CV risk [7]. This was supported by extensive evidence that both elevated triglycerides and low HDL cholesterol were predictive for CVD, independent of LDL cholesterol concentration. Subsequent guidelines and expert consensus have recognised the importance of atherogenic dyslipidaemia as a key driver of CV risk in insulin-resistant states, even if LDL cholesterol levels are well controlled [8-12]. Thus, targeting atherogenic dyslipidaemia secondary to LDL cholesterol reduction has the potential for reducing this risk. Five years on, the key question must be: Are we closer to defining the optimal therapeutic strategy for reducing lipid-related residual CV risk?

### New insights: what is the relevance of HDL versus TRLs to residual risk?

Emerging evidence provides new insight into the relative importance of TRLs versus HDL cholesterol as a driver of residual CV risk. Without doubt, the epidemiological data for low HDL cholesterol as an important conditional CV risk factor is robust, supported by evidence from the Prospective Cardiovascular Münster (PROCAM) study, and the Emerging Risk Factors Collaboration [13,14]. Furthermore, there is clear evidence from animal studies that HDL-raising interventions inhibit the development of atherosclerosis [15]. These data support the integration of plasma HDL cholesterol concentration into the PROCAM risk score and SCORE charts for CV risk assessment [8,14,16]. However, it is acknowledged that the genetic evidence in support of a protective role of HDL in humans is more contentious. A recent analysis using a Mendelian randomisation approach showed that lifelong exposure to higher plasma levels of HDL cholesterol among carriers of the loss-of-function endothelial lipase genetic variant (*LIPG Asn396Ser*) did not translate to reduction in myocardial infarction (MI) risk. Furthermore, there was no association between an increase in HDL cholesterol levels according to genetic score (based on 14 variants associated with HDL cholesterol) and risk for MI, whereas there was concordance for LDL cholesterol [17]. These data appear to challenge the importance of HDL cholesterol as a driver of residual CV risk, a contribution further confounded by the presence of different HDL subclasses.

However, we need to take a step back and consider the relevance of HDL cholesterol concentration to the atheroprotective capacity of HDL. Experimental studies support a number of biological activities of HDL with potential for atheroprotection [18]. Perhaps the most important is the ability of HDL to promote cholesterol efflux from cells, including from macrophages in the arterial wall. As peripheral cholesterol efflux contributes less than 5% of the cholesterol content of HDL, it would be reasonable to assume that HDL cholesterol concentration may be a poor surrogate measure for estimating reverse cholesterol transport, HDL function and CV risk [19]. A preferable approach may involve measurement of the concentration of specific HDL particle subclasses [20,21], given evidence that these exhibit a number of biological activities that might be relevant to the pathophysiology of atherosclerosis. However, it is recognised that these are static measurements that may not accurately reflect the dynamic nature of HDL particle populations.

Latest thinking is that HDL functionality may be more important than HDL quantity. Indeed, the relevance of this approach is underlined by in vitro evidence that the potential atheroprotective capacity of HDL is impaired in certain disease states, including stable and unstable coronary heart disease (CHD), as well as diabetes [22-25]. In the latter setting, prolonged hyperglycaemia has been associated with the formation of HDL that are defective in anti-inflammatory activity [22]. Thus, from a therapeutic perspective, improving the functionality of HDL, as well as raising plasma HDL cholesterol levels, might represent a more complementary approach. However, our limited understanding of the complexity of HDL particles, which are heterogeneous in terms of origin, size, composition, structure and biological function, has so far hampered efforts to define a suitable marker of HDL functionality, let alone translate such measures to the clinical setting. It is also a prerequisite that functionality must parallel plasma concentration of HDL in the general population, to explain the robust association of HDL cholesterol levels with CV risk. However, every means of raising HDL cholesterol levels may not necessarily increase HDL functionality.

There has also been re-evaluation of the importance of TRLs as a driver of residual CV risk, especially in the context of cardiometabolic disease. Evidence supports a long-standing association between the level of triglycerides (including nonfasting triglycerides), and CVD [26,27]. In addition, genetic studies show that variants associated with triglyceride-related pathways (for example the *APOA5* variant *1131T>C*), are associated with coronary risk [28]. However, in the Emerging Risk Factors Collaboration [13] the relationship between plasma triglycerides concentration and CVD was either attenuated or abolished after adjusting for other risk factors. These conflicting data may be explained by the view that triglycerides *per se* are not atherogenic but instead represent a marker of CV risk because of their association with atherogenic TRLs and their remnants [10,29,30]. In fact, there is important heterogeneity in TRL particles in terms of size, composition and atherogenicity. Experimental studies have shown that elevated plasma levels of TRLs and their remnants, especially during the postprandial phase, accentuate inflammatory responses, thereby increasing endothelial dysfunction [29,30], and may act to suppress the atheroprotective and anti-inflammatory effects of HDL [31,32]. Elevated levels of TRL remnant cholesterol also contribute directly to plaque formation and progression [33].

**Figure 2:**
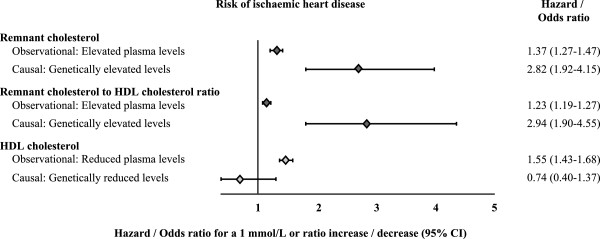
**Remnant cholesterol, estimated indirectly as total cholesterol minus the cholesterol contents of LDL and HDL, was shown to be causal for ischaemic heart disease, independent of HDL cholesterol.** Reproduced with permission from Varbo et al. [34].

A recent study has provided evidence of a causal association between remnant cholesterol contained in TRLs and ischaemic heart disease [34]. A Mendelian randomisation design was used to overcome confounding between remnant cholesterol and other risk factors including HDL, a major flaw in previous observational studies [35,36]. The genes studied were those affecting levels of HDL, LDL and triglycerides. In this study, a 1 mmol/L (39 mg/dL) increase in estimated levels of non-fasting remnant cholesterol (defined as total cholesterol – [cholesterol in LDL and HDL]) was associated with a 2.8-fold causal risk for ischaemic heart disease; this was double the risk based on observational data alone (hazard ratio 1.4, 95% confidence interval [CI] 1.3 to 1.5) [34]. These findings imply that lifelong exposure to TRLs through genetically elevated remnant cholesterol levels may have a larger effect on coronary risk. In contrast, there was no association between HDL cholesterol concentration and risk for ischaemic heart disease (Figure 2). However, it is not clear whether the data would have been as strong if non-HDL triglycerides (albeit more complicated to measure) had been used. Even with this caveat, this study highlights the importance of remnant cholesterol contained in TRLs as a key contributor to residual CV risk.

In conclusion, given the metabolic interrelationships between HDL and TRL-related pathways, and synergistic effects on CV risk when both components of atherogenic dyslipidaemia are present, even if LDL cholesterol is at goal [37,38], the R^3^i believes that targeting both lipid abnormalities is a key approach to reducing lipid-related residual CVD risk. In this context, it is relevant that the updated PROCAM risk score includes both HDL cholesterol and triglycerides, a marker of TRLs, thereby recognising the importance of both factors to residual CV risk.

### Assessment of residual CV risk

The previous mechanistic insights highlight the need for lipid/lipoprotein targets that better reflect the burden of atherogenic dyslipidaemia. Issues regarding the relevance of HDL cholesterol concentration have already been raised, but in the absence of validated data for HDL functionality, no alternatives exist. In individuals with insulin-resistant conditions and elevated triglycerides, current guidelines and the recent International Atherosclerosis Society (IAS) Position Paper recommend non-HDL cholesterol as the preferred target [8,39]. By definition, non-HDL cholesterol essentially represents the sum of cholesterol in LDL cholesterol and very low-density lipoprotein (VLDL) cholesterol; the latter is regarded as increasingly important as a driver of residual CV risk. Moreover, non-HDL cholesterol can be measured in non-fasting serum. Consistent with the IAS, the R^3^i strongly believes that there should be renewed emphasis on the use of non-HDL cholesterol as a key target for treatment decisions relating to lipid-related residual CV risk.

An alternative approach may be to consider atherogenic dyslipidaemia as a continuous variable using the ratio log(triglycerides)/HDL cholesterol or log(triglycerides/HDL cholesterol), based on fasting measures. In recent studies this ratio was associated with a stepwise increase in residual coronary risk, and was also predictive of poorer metabolic control in people with type 2 diabetes [40-42]. However, taking into account the evidence base and practical issues in lipid analysis, measurement of non-HDL cholesterol is the simplest, most pragmatic target for therapeutic strategies.

### Targeting residual risk: current approaches

Lifestyle intervention underpins the management of atherogenic dyslipidaemia, as reinforced in recent guidelines [8]. However, it is recognised that long-term adherence to a healthy lifestyle is frequently problematic. Additionally, even among highly-motivated individuals, intensive therapeutic lifestyle intervention may be insufficient to reduce CV risk. Recent findings from the Look AHEAD trial illustrate this [43]. Among obese/overweight individuals with type 2 diabetes, intensive lifestyle intervention involving both dietary and physical activity measures, did not significantly reduce major CV events beyond that observed with diabetes support and education alone. It is, however, acknowledged that intensive lifestyle intervention did significantly impact atherogenic dyslipidaemia, on average raising HDL cholesterol by ~5 mg/dL (0.13 mmol/L) and reducing fasting triglycerides by ~30 mg/dL (0.34 mmol/L) at the end of the 10-year study period. In addition, subjects in the intensive lifestyle group had better control of diabetes and blood pressure than those in the comparator group, despite reductions in their medications. These data clearly provide justification for lifestyle intervention as the first step for managing atherogenic dyslipidaemia, but also indicate that most high-risk patients will also require pharmacotherapy.

#### Current pharmacotherapeutic strategies

While definitive support for therapeutic options targeting atherogenic dyslipidaemia to reduce residual CV risk is still awaited, recent trials of fibrates, niacin (nicotinic acid), omega-3 fatty acids and ezetimibe provide insights.

##### Fibrates

**Figure 3:**
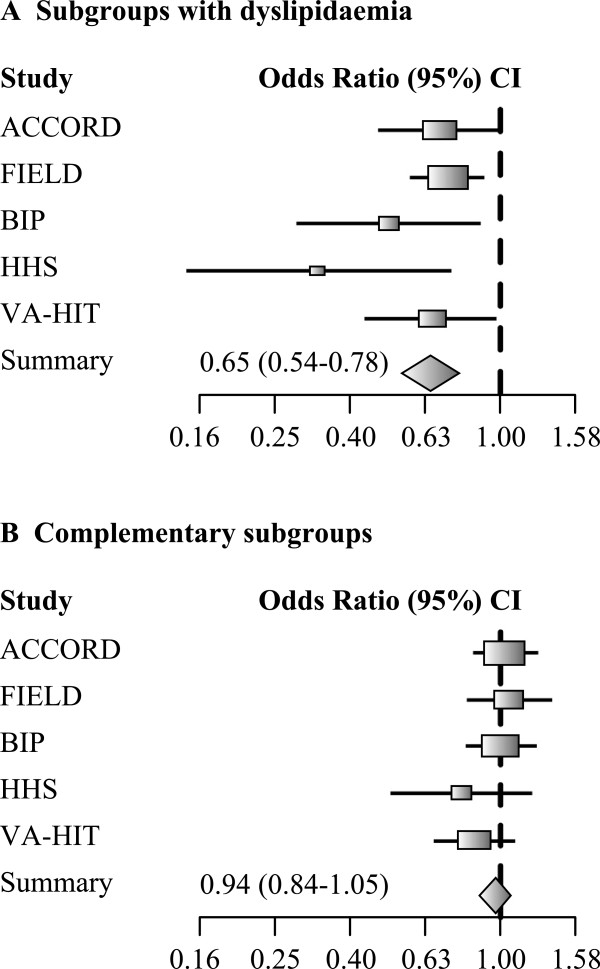
**Meta-analysis of major fibrate outcomes studies, showing the impact of fibrate treatment on residual CV risk in patients with atherogenic dyslipidaemia.** An odds ratio <1 indicated a beneficial therapeutic effect. The two panels show data from subgroups of patients with dyslipidaemia i.e., high levels of triglycerides and low levels of high-density lipoprotein [HDL] cholesterol, **Panel A**; or from the complementary subgroups without this dyslipidaemia, **Panel B**. Subgroups with dyslipidaemia defined according to the ACCORD Lipid trial (triglycerides ≥204 mg/dL or 2.3 mmol/L and HDL cholesterol ≤34 mg/dL or 0.9 mmol/L) or closest to these lipid criteria in each of the other trials were used in this analysis. The outcome defined for each individual trial was used. A total of 2,428 fibrate-treated subjects (302 events) and 2,298 placebo-treated subjects (408 events) with dyslipidaemia were included in the analysis reported in **A**. Reproduced with permission from Sacks et al. [47].

Fibrates (peroxisome proliferator-activated receptor-α [PPARα] agonists) represent one option, especially in the context of high-risk individuals with insulin-resistant conditions [44]. In subgroup analyses of the FIELD (Fenofibrate Intervention and Event Lowering in Diabetes) study, type 2 diabetes patients with marked atherogenic dyslipidaemia, defined as triglycerides ≥204 mg/dL (2.3 mmol/L) and low HDL cholesterol, obtained greater benefit from fenofibrate treatment than those without this dyslipidaemia. In this group, which comprised 19% of the total study population, there was a 27% relative reduction in CV risk (versus 11% in all patients) [45]. Subsequently, in the ACCORD (Action to Control Cardiovascular Risk in Diabetes) Lipid Trial (n = 5,518), a pre-defined subgroup analysis indicated substantial benefit associated with combining fenofibrate with background simvastatin in type 2 diabetes patients with marked atherogenic dyslipidaemia defined by baseline triglycerides in the upper third of the population (≥204 mg/dL or 2.3 mmol/L) and baseline HDL cholesterol levels in the lower third (≤34 mg/dL or 0.9 mmol/L). This group represented about 17% of the overall study population. In these patients, there was ~30% relative reduction in CV events versus no benefit in 83% of the study population without this dyslipidaemia (Table 1) [46]. Furthermore, a meta-analysis of subgroups with similar lipid criteria for atherogenic dyslipidaemia in the major fibrate trials confirmed this benefit on residual CV risk (Figure 3) [47]. Fibrate treatment was associated with a 35% relative reduction in CV risk in individuals with atherogenic dyslipidaemia versus 6% in individuals without this dyslipidaemia. A subsequent meta-analysis of fibrate trials (n = 45,058) indicated that the reduction in CV risk associated with this therapy class is predominantly due to prevention of coronary events [48]. The observed benefits of triglyceride-lowering on residual CV risk may relate to the effects of endogenous pathways for PPARα activation on atherosclerosis. Lipolysis in certain situations can generate PPAR ligands and also limit some known inflammatory responses [49]. Indeed, recent genome-wide association studies show that gain of function variants of the lipoprotein lipase gene, coding for the enzyme which hydrolyses the triglyceride core of plasma chylomicrons and VLDL to release free fatty acids, are associated with a decrease in plasma triglycerides and coronary risk [50].

**Table 1:** Recent trials investigating effects of fibrates, niacin or omega-3 fatty acids added to statin on residual cardiovascular risk

**Treatment/Trial [reference]**	**Daily dose (mg)**	**Patient criteria**	**Duration of follow-up**	**Key findings**
Fenofibrate				
ACCORD Lipid (n = 5,518) [46]	160^1^	Type 2 diabetes; Mean LDL-C ~2.07 mmol/L [80 mg/dL] on simvastatin (mean dose 22.4 mg/day)	4.7 years	• No significant benefit on any CV outcomes for the total study population
				• For patients with marked atherogenic dyslipidaemia,^2^ there was ~30% reduction in the primary CV outcome versus simvastatin alone (12.4% versus 17.3%, p = 0.06 for interaction versus all other patients)
Niacin				
AIM-HIGH (n = 3,414) [61,62]	ER niacin titrating to 1500-2000	Patients with CVD with persistent atherogenic dyslipidaemia^3^	Prematurely terminated; mean 3 years	• No significant outcomes benefit with ER niacin
				• Methodological issues; inadequately powered, placebo contained a low-dose of niacin (50 mg/capsule), imbalance in concomitant LDL-C lowering therapy between groups
		Median LDL-C on statin 1.91 mmol/L [74 mg/dL]		
				• For patients with marked atherogenic dyslipidaemia,^4^ there was a 36% relative reduction in the primary CV outcome (25.0% versus 16.7%, p = 0.032)
HPS2-THRIVE (n = 25,673) [63]	ER niacin/laropiprant 2000	Patients with history of CVD	Median 3.9 years	• No significant outcomes benefit with ER niacin/laropiprant
		Mean lipid values at end of pre-randomisation phase (simvastatin 40 mg/day ± ezetimibe):		
				• Significant increases in diabetic complications, new-onset diabetes, infection, gastrointestinal effects (p < 0.0001), musculoskeletal, bleeding effects (p < 0.001) and skin adverse events (p = 0.026) with ER niacin/laropiprant
		LDL-C 1.64 mmol/L [63 mg/dL]		
		HDL-C 1.14 mmol/L [44 mg/dL]		
		TG 1.43 mmol/L [125 mg/dL]		
Omega-3 fatty acids				
JELIS (n = 18, 645) [65,66]	1800, EPA	High-risk patients with hypercholesterolaemia (total cholesterol ≥6.5 mmol/L [250 mg/dL])	Mean 4.6 years	• 19% reduction in major coronary events (2.8% versus 3.5%, p = 0.011) with EPA + statin versus statin alone • A post hoc analysis showed a 53% relative reduction (p = 0.043) in patients with TG ≥1.7 mmol/L (150 mg/dL) and HDL-C <1.0 mmol/L (40 mg/dL) versus those without this dyslipidaemia
		Baseline mean LDL-C 4.6 mmol/L [180 mg/dL] on pravastatin 10 mg/day or simvastatin 5 mg/day		
ALPHA-OMEGA (n = 4,837) [67]	400, EPA + DHA;	MI survivors, 85% on lipid-lowering therapy (mainly statins)	40 months	• No significant effect on CV outcomes with any treatment versus placebo (best evidence-based treatment)
	2 g ALA; or both			
		Mean baseline lipids were		
		LDL-C 2.6 mmol/L [100 mg/dL]		
		HDL-C 1.28 mmol/L [49.5 mg/dL]		
		Median TG 1.69 mmol/L [150 mg/dL]		
ORIGIN	900 (465 EPA and 375 DHA)	Patients with or at risk of diabetes and at high CV risk	Median 6.2 years	• No significant effect on primary outcome (CV death) or secondary or other clinical outcomes
(n = 12,536) [68]				
		Mean baseline lipids were		
		TC 4.9 mmol/L (190 mg/dL)		
		LDL-C 2.89 mmol/L (112 mg/dL)		
		HDL-C 1.19 mmol/L (46 mg/dL)		
		Median TG 1.58 mmol/L (140 mg/dL)		

^1^Dose at start of trial, subsequently adjusted according to estimated glomerular filtration rate using the abbreviated Modification of Diet in Renal Disease equation; ^2^Marked atherogenic dyslipidaemia defined as baseline triglycerides in the upper third of the population (≥204 mg/dL or 2.3 mmol/L) and baseline HDL cholesterol levels in the lower third (≤34 mg/dL or 0.9 mmol/L); ^3^Atherogenic dyslipidaemia defined as median HDL-C 0.91 mmol/L [35 mg/dL] and median triglycerides 1.82 mmol/L [161 mg/dL]; ^4^Marked atherogenic dyslipidaemia defined as triglycerides >200 mg/dL or 2.3 mmol/L and HDL-C <32 mg/dL or 0.83 mmol/L.

ACCORD Action to Control Cardiovascular Risk In Diabetes; AIM-HIGH Atherothrombosis Intervention in Metabolic Syndrome With Low HDL/High Triglycerides: Impact on Global Health Outcomes; HPS2-THRIVE Heart Protection Study 2-Treatment of HDL to Reduce the Incidence of Vascular Events; JELIS Japan Eicosapentaenoic acid Lipid Intervention Study; ORIGIN Outcome Reduction with an Initial Glargine Intervention; ALA alpha-linolenic acid; CV cardiovascular; CVD cardiovascular disease; DHA docosahexaenoic acid EPA eicosapentaenoic acid; ER extended-release; HDL-C high-density lipoprotein cholesterol; LDL-C low-density lipoprotein cholesterol; MI myocardial infarction; TC total cholesterol; TG triglycerides.

An ancillary study of the ACCORD Lipid trial (supported by the R^3^i), which evaluated effects on postprandial lipaemia, provides a possible explanation as to why the benefit of fibrates is specific to patients with atherogenic dyslipidaemia [51]. Compared with the simvastatin monotherapy group, treatment with the combination of fenofibrate plus simvastatin led to significant reductions in postprandial exposure to triglycerides and apoB48, indicating a decrease in the accumulation of atherogenic, intestinally-derived TRL remnants. This effect of fenofibrate was selective to patients with elevated fasting triglycerides at baseline. Added to this, there is also evidence of benefit with fenofibrate on diabetes-related microvascular complications, including diabetic retinopathy and nephropathy [52-55].

Despite this favourable profile of macro- and microvascular benefits, the efficacy of the current fibrates may be limited by uncertainties relating to the optimal level of PPARα-agonism, agonist-specific biologic responses, and the side effects of current synthetic agonists. Of relevance for patients already on statin therapy, is the increased risk of muscle-related symptoms with the combination of a statin and certain fibrates, specifically gemfibrozil. This does not appear to be an issue with fenofibrate, probably due to the lack of interference of fenofibrate on statin pharmacokinetics [8,56]. Indeed, there was no adverse signal for myopathy in the ACCORD Lipid trial [44]. Other commonly reported adverse effects include increases in homocysteine, creatinine and liver enzymes, markers of CVD, renal disease and hepatic dysfunction, respectively [46,54]. In the ACCORD Lipid study, 48% of statin-treated patients who received the full dose of fenofibrate (160 mg/day) for at least 30 days before the 4-month follow-up showed >20% increase from baseline in serum creatinine (versus 9% in the placebo group) at this visit [57]. Both the ACCORD Lipid and the FIELD studies subsequently showed that increases in serum creatinine associated with fenofibrate were reversible, with levels returning to those observed in the placebo group about 6–8 weeks after cessation of treatment, and did not appear to detrimentally influence risk for CVD or deterioration in renal function [58-60]. In the FIELD study, despite increasing creatinine levels, fenofibrate treatment was associated with less renal function decline, as shown by a slower rate of loss of estimated glomerular filtration rate over 5 years compared with placebo [59]. However, in high-risk older patients with impaired renal function receiving multiple treatments likely to affect renal haemodynamics, this may be a relevant consideration.

##### Niacin

Recent trials with niacin (nicotinic acid) have been disappointing (Table 1). The AIM-HIGH (Atherothrombosis Intervention in Metabolic Syndrome With Low HDL/High Triglycerides: Impact on Global Health Outcomes) trial (n = 3,414) [61] evaluated the effect of extended-release (ER) niacin (1.5-2 g/day) on residual CV risk in patients with CVD and optimally treated with a statin but with residual atherogenic dyslipidaemia (median HDL cholesterol 35 mg/dL or 0.91 mmol/L and median fasting triglycerides 161 mg/dL or 1.82 mmol/L). The study was terminated 18 months earlier than planned due to futility. A number of factors may explain the lack of benefit. First, because of limited financial support, the small study cohort required a very large effect size of 25% benefit for statistical significance. Second, the trial design was flawed by the inclusion of a low dose of niacin (50 mg/capsule) in the placebo, which may have contributed to the 12% increase in HDL cholesterol plasma concentration in the control group. Third, there was imbalance in concomitant LDL lowering therapy between the two groups; in the placebo group, 75% of patients received simvastatin 40 mg/day or higher and 21% also received add-on ezetimibe. Despite these limitations, a subgroup analysis of AIM-HIGH in individuals with triglycerides >200 mg/dL (2.3 mmol/L) and HDL cholesterol <32 mg/dL (0.83 mmol/L) showed a 36% relative reduction (p = 0.032) in the primary composite end point, consistent with findings from the fibrate meta-analysis [47,62].

More recently, the much larger HPS2-THRIVE (Heart Protection Study 2-Treatment of HDL to Reduce the Incidence of Vascular Events) (n = 25,673) [63] failed to show a benefit on clinical outcomes with a different niacin formulation (combined with laropiprant, which attenuates the niacin flushing response). However, it should be borne in mind that at baseline the HPS2-THRIVE patient population had very well controlled LDL cholesterol levels (mean 63 mg/dL or 1.64 mmol/L) and was outside of the thresholds used to define atherogenic dyslipidaemia (mean HDL cholesterol was 44 mg/dL or 1.14 mmol/L, and triglycerides were 125 mg/dL or 1.43 mmol/L). Consequently the trial did not test the effect of niacin on residual CV risk due to atherogenic dyslipidaemia. Moreover, there were safety issues with niacin/laropiprant, notably significant increases in diabetes complications, new-onset diabetes, infections, and gastrointestinal, musculoskeletal, bleeding and skin adverse events, leading to subsequent world-wide withdrawal of this therapy [64]. Niacin remains a therapeutic option in North and South America, but is not an option in Europe following suspension of niacin/laropiprant by the European Medicines Agency.

##### Omega-3 fatty acids

With respect to omega-3 fatty acids, there were positive findings from JELIS (Japan Eicosapentaenoic acid Lipid Intervention Study) (n = 18,645) with the combination of omega-3 fatty acids (1.8 g/day of eicosapentaenoic acid [EPA]) plus statin versus statin alone, both overall and in patients with the metabolic syndrome (Table 1) [65]. A *post hoc* analysis of JELIS evaluated the effect of EPA treatment in individuals with atherogenic dyslipidaemia as defined by triglycerides ≥150 mg/dL (1.7 mmol/L) and HDL cholesterol <40 mg/dL (1.0 mmol/L), representing 5% of the total study population. In this dyslipidaemic group, EPA treatment reduced the risk of coronary artery disease by 53% (p = 0.043), compared with individuals without this dyslipidaemia [66].

In contrast, the Alpha-Omega trial (n = 4,837) failed to show a benefit on major CV events with the addition of n−3 fatty acids (margarine containing EPA plus docosahexaenoic acid with a targeted additional daily intake of 400 mg) in patients with a previous MI receiving best evidence-based antihypertensive, antithrombotic, and lipid-modifying therapy (mainly statins) [67]. More recently, the ORIGIN (Outcome Reduction with an Initial Glargine Intervention) trial [68] used a 2x2 factorial design to investigate the effect of treatment with omega-3 fatty acids (1 g capsule containing ≥900 mg of ethyl esters of n-3 fatty acids) versus placebo (one arm) and insulin glargine versus standard care (second arm) in subjects with or at high risk for diabetes and at increased CV risk (n = 12,536). Treatment with n-3 fatty acids had no significant effect on the primary outcome (CV death) or any of the secondary or additional outcomes. It has been suggested that the lack of effect may relate to the selected dose, and/or extensive use of concomitant cardioprotective treatments. Furthermore, a recent meta-analysis of trials of omega-3 supplementation (n = 68,680 subjects in 20 trials, mean dose 1.51 g/day) failed to show any significant association with all-cause mortality, cardiac death, sudden death, MI, or stroke [69]. There is also some evidence from recent trials to suggest a possible link between high plasma concentrations of omega-3 fatty acids and increased risk of prostate cancer [70,71].

A new formulation of omega-3 fatty acids (AMR101, ≥96% EPA ethyl ester) is being evaluated in the REDUCE-IT trial in statin-treated patients with elevated trigycerides (>1.7 mmol/L or 150 mg/dL) and at least one CV risk factor [72]. However, results are not expected until 2017.

##### Ezetimibe

Combination therapy with statin plus ezetimibe may represent an alternative approach to the management of residual CV risk [10]. In a meta-analysis of 27 trials (>21,000 patients), there was incremental lowering of LDL cholesterol (by 15%), non-HDL cholesterol (by 13%) and triglycerides (by 5%), and raising of HDL cholesterol (by 1.6%) with the combination of ezetimibe plus statin compared with statin alone [73]. However, so far there are limited outcomes data to support this strategy. Although there was significant reduction in major atherosclerotic events (coronary death, MI, ischaemic stroke, or any revascularisation procedure) in patients with advanced renal disease in SHARP (Study of Heart and Renal Protection), SEAS (Simvastatin and Ezetimibe in Aortic Stenosis) failed to show significant clinical benefit in patients with aortic stenosis [74,75]. Additionally, in the ENHANCE (Ezetimibe and Simvastatin in Hypercholesterolemia Enhances Atherosclerosis Regression) trial, treatment with the combination of simvastatin plus ezetimibe did not result in a significant difference in changes in intima-media thickness compared with simvastatin alone in patients with familial hypercholesterolaemia. This was despite significant reductions from baseline in LDL cholesterol by 55% and triglycerides by 30%, and an increase in HDL cholesterol by 10% in the combination therapy group [76].

It may be that treatment with ezetimibe for 2 years is insufficient to differentiate significant incremental clinical benefit, against a background of lipid-modifying and pleiotropic effects of statin therapy. In support, while partial ileal bypass surgery in POSCH (Program on the Surgical Control of the Hyperlipidemia) and statin treatment in 4S (Scandinavian Simvastatin Survival Study) produced similar LDL cholesterol lowering (37.7% versus 35%), the separation of curves for CV outcomes of treated versus non-treated subjects was evident after about 4.5 years in POSCH but only 1.5 years in 4S [77,78]. However, the possibility of pleiotropic effects with ezetimibe cannot be discounted, on the basis of recent findings [79]. Results from IMPROVE-IT (IMProved Reduction of Outcomes: Vytorin Efficacy International Trial) [80] comparing statin-ezetimibe combination therapy versus statin alone against a background of best evidence-based treatment in the acute coronary syndrome (ACS) setting are critical to resolve this issue.

### Do emerging therapies offer new hope?

While targeting atherogenic dyslipidaemia with the addition of a fibrate or niacin may reduce residual CV risk in statin-treated patients by about one-third, it is clear that additional therapeutic options are also needed. Among emerging therapies, there are a number of novel approaches that may offer potential benefit (Table 2) [81-88].

**Table 2:** Emerging treatments with potential for targeting atherogenic dyslipidaemia

		Effects on atherogenic dyslipidaemia*		**Other effects**	**Outstanding issues**
**Treatment group/example**	**Dose**	**Triglycerides**	**HDL-C**		
**[reference]; duration**					
SPPARMs: K-877 [81]	100 μg BID	↓70%	↑18%	Improved safety profile (CV, renal and hepatic biomarkers) versus fenofibrate	● Outcomes data, long-term safety
12 weeks					
Dual PPAR agonists					
GFT505 (dual PPARα/δ) [84]	80 mg OD	↓17-25%	↑8-9%	Improved safety profile (hepatic biomarkers)	● Lower efficacy than current PPARα agonists
4 weeks					● Outcomes data, long-term safety

CETP inhibitors					
Anacetrapib^1^[85]	100 mg OD	↓7%	↑138%	Decreases in LDL-C (~40%), Lp(a) and apoB	● Outcomes data, long-term safety
24 weeks					
Evacetrapib^1^[86]	100 mg OD	NA	↑~80%	Decreases in LDL-C (36%); data on other lipid effects NA	● Outcomes data, long-term safety
12 weeks					
PCSK9 targeted therapy					
Alirocumab^1^[87]	150 mg every 2 weeks	↓19%	↑6%	Decreases in LDL-C (>60%); also Lp(a) and apoB	● Outcomes data, long-term safety
12 weeks					
AMG 145^1,2^[88]	140 mg every 2 weeks	↓34%	↑8%	Decreases in LDL-C (>60%), Lp(a) and apoB	● Outcomes data, long-term safety
12 weeks					

*Placebo-adjusted effect; ^1^Change from baseline in combination with a statin; ^2^AMG 145 is now referred to as evolocumab BID twice daily; apo apolipoprotein; CETP cholesteryl ester transfer protein; CV cardiovascular; LDL-C low-density lipoprotein cholesterol; Lp(a) lipoprotein(a); NA not available; OD once daily; PCSK9 proprotein convertase subtilisin/kexin type 9; PPAR peroxisome proliferator-activated receptor; SPPARMs Selective peroxisome proliferator-activated receptor modulators.

#### Next generation PPAR agonists

One area of interest is the next generation of PPAR agonists. There are three PPAR isoforms with different pharmacological activities; PPARα plays a key role in lipid metabolism, whereas PPARγ and PPARδ are critical players in regulating energy metabolism in adipose tissue and muscle, as well as being targets for the treatment of insulin resistance. Thus, it was proposed that dual PPAR agonists with selective activity for PPARα/γ or PPARα/δ may offer opportunities to concomitantly manage several areas of dysmetabolism, especially in the context of cardiometabolic disease [89]. However, the recent termination of aleglitazar, a dual PPARα/γ agonist, due to adverse safety signals and lack of efficacy based on the recommendation of the Independent Data and Safety Monitoring Board of the AleCardio (Aleglitazar in Patients With a Recent Acute Coronary Syndrome and Type 2 Diabetes Mellitus) phase III trial is a disappointment for this therapeutic class [90]. Previously, aleglitazar showed favourable effects on glucose homeostasis and atherogenic dyslipidaemia in the phase II SYNCHRONY trial in type 2 diabetes patients, although at all doses (50 μg, 150 μg, 300 μg, or 600 μg once daily) there were dose-dependent increases in body weight and the number of patients developing oedema [82]. Saroglitazar is currently the only agent of this class approved for the treatment of type 2 diabetes (India, June 2013).

Dual PPARα/δ agonists are also under investigation; the most advanced in development is GFT505, which has preferential activity on PPARα [89]. In a phase II trial in patients with combined dyslipidaemia or prediabetes, GFT505 improved glucose homeostasis, reduced triglycerides and LDL cholesterol, and raised HDL cholesterol levels [84,91]. GFT505 also reduced liver enzymes, suggesting potential for the management of individuals with prediabetes and non-alcoholic fatty liver disease [91].

In addition, K-877, a highly potent and selective PPARα modulator (SPPARM), is undergoing phase II/III development for the management of atherogenic dyslipidaemia. Mechanistically, K-877 offers advantages over fenofibrate in terms of increased PPARα potency and improved PPAR safety profile (CV, renal and hepatic biomarkers) [89]. In a phase II trial, K-877 showed improved lipid-modifying efficacy and greater effects on postprandial lipaemia compared with fenofibrate, in particular suppressing the postprandial increase in triglycerides and atherogenic intestinal (apoB48) remnant cholesterol [81,92,93]. There was also evidence suggestive of an improved safety profile with K-877 over fenofibrate [81].

#### Cholesteryl ester transfer protein (CETP) inhibitors

Cholesteryl ester transfer protein (CETP) inhibition has also attracted attention as a therapeutic approach. CETP has a critical role in the intravascular mass transfer and heteroexchange of cholesteryl ester and triglycerides between HDL and TRL and their remnants in vivo. Thus, CETP inhibition may be anti-atherogenic by increasing the concentration of cholesterol in the HDL fraction, and/or by decreasing LDL or the cholesterol content of TRL. There have been issues with the first two CETP inhibitors, torcetrapib and dalcetrapib. The former was terminated due to safety issues in ILLUMINATE (Investigation of Lipid Level Management to Understand Its Impact in Atherosclerotic Events), probably due to off-target effects on blood pressure and/or the artery wall [94,95]. In contrast, dalcetrapib was terminated due to futility in dal-OUTCOMES in ACS patients. The reason for this is uncertain, but may relate to the patient population, given the lack of association between baseline HDL cholesterol and the risk of incident CV events observed in the placebo group [96].

The two CETP inhibitors that are currently most advanced in development, anacetrapib and evacetrapib, have so far shown no adverse safety signal in phase II trials, and have beneficial effects in raising HDL cholesterol, as well as lowering triglycerides and atherogenic lipoproteins including LDL cholesterol and lipoprotein(a) [85,86]. Both agents are now under study in major prospective outcomes studies – REVEAL (Randomized EValuation of the Effects of Anacetrapib Through Lipid-modification) with anacetrapib and ACCELERATE (A Study of Evacetrapib in High-Risk Vascular Disease) with evacetrapib – to evaluate whether their lipid-modifying effects translate to reduction in CV risk beyond that observed with LDL cholesterol lowering with a statin [97,98].

#### PCSK9-targeted therapy

There is considerable interest in novel therapy targeting proprotein convertase subtilisin/kexin type 9 (PCSK9), which has a pivotal role in promoting degradation of hepatic LDL receptors, and hence in LDL homeostasis. Thus, inhibition of PCSK9 prevents LDL receptor degradation and offers the possibility of lowering LDL cholesterol levels, a rationale which is now supported by clinical trials [99].

Indeed, monoclonal antibody therapy targeting PCSK9 holds promise. Both of the most advanced therapies, alirocumab and evolocumab (AMG 145), have been shown to improve attainment of LDL cholesterol goals in high-risk statin-treated patients, and have beneficial effects on other lipids, including lowering atherogenic TRLs and lipoprotein(a), and modestly raising HDL cholesterol [87,88]. Although these trials have been relatively short-term to date, there is no evidence yet to suggest any significant adverse signal. Major phase III prospective trials are now in progress to assess their long-term safety and effects on residual CV risk in high-risk statin-treated patients [100,101].

#### ApoA-I therapies

Finally, the development of novel apoA-I therapies may have potential application in managing residual CV risk in the ACS setting. The rationale for this approach is supported by evidence of reduction in coronary atherosclerosis in ACS patients following infusion of recombinant apoA-I_Milano_[102]. In experimental models, recent apoA-I mimetics were shown to have atheroprotective effects, including anti-inflammatory properties [103]. In phase I studies, infusion of CSL112, a novel formulation of reconstituted apoA-I, improved prebeta1-HDL, as well as global cholesterol efflux capacity from macrophages, and had strong anti-inflammatory activity [104]; this agent is now in phase II development. RVX-208, which acts via an epigenetic mechanism to increase apoA-I synthesis, did not meet its primary outcome (0.6% change in percent atheroma volume) in the ASSURE (ApoA-I Synthesis Stimulation and Intravascular Ultrasound for Coronary Atheroma Regression Evaluation) trial in patients with angiographic evidence of CHD and low HDL cholesterol [105]. It is recognised that these agents are at an early stage of development and have not been tested in outcomes studies in ACS patients.

### Conclusion: looking to the future for residual CV risk

The management of residual CV risk is a major challenge for the 21st century, compounded by the escalating pandemics of obesity, metabolic syndrome and type 2 diabetes. As discussed, atherogenic dyslipidaemia – the combination of elevated TRLs and their remnants and low HDL – is an important modifiable contributor to residual CV risk. There is general consensus regarding the importance of atherogenic dyslipidaemia as a key driver of CV risk in individuals with cardiometabolic disease [8,12]. However, the recently published American College of Cardiology/American Heart Association (ACC/AHA) Guidelines on the Treatment of Blood Cholesterol [106] solely focus on statins for CVD prevention, and omit consideration of other therapies for management of residual CV risk in high-risk patients with persistent atherogenic dyslipidaemia despite being at LDL cholesterol goal. In the context of the escalating burden of obesity and type 2 diabetes, the ACC/AHA guidelines are clearly an oversimplification of dyslipidaemia management. Given that these guidelines ignore both the strength and congruence of the evidence from clinical and mechanistic studies for atherogenic dyslipidaemia, it is not surprising that both the US National Lipid Association and the American Association of Clinical Endocrinologists have failed to endorse the new ACC/AHA guidelines [107,108]. The European Atherosclerosis Society (EAS) also reaffirms that the European Society of Cardiology/EAS guidelines for management of dyslipidaemia are more appropriate in Europe [109].

Yet even where evidence-based guidelines recognise and incorporate management strategies for atherogenic dyslipidaemia, there is still lack of awareness in routine practice. For example, the Dyslipidemia International Study (DYSIS) in 22,063 statin-treated outpatients in Europe and North America showed that the prevalence of elevated triglycerides and/or low HDL cholesterol was highest (exceeding 40%) in high-risk individuals [110]. Clearly there is an urgent need for action to address this unmet challenge.

As part of its mission to improve the awareness and clinical management of atherogenic dyslipidaemia to reduce residual CV risk, the R^3^i highlights a number of priorities. The R^3^i issues four recommendations: (i) education is needed to improve awareness of atherogenic dyslipidaemia as a key driver of lipid-related residual cardiovascular risk, especially in high-risk patients with insulin-resistant conditions; (ii) non-HDL cholesterol is the preferred target for treatment decisions relating to atherogenic dyslipidaemia; (iii) the addition of a fibrate, niacin (North and South America), omega-3 fatty acids or ezetimibe to statin therapy are approaches to reduce non-HDL cholesterol. Post hoc analyses indicate that addition of a fibrate or niacin can reduce residual CV risk by about one-third in high-risk statin-treated patients with atherogenic dyslipidaemia; and (iv). Additional approaches are clearly needed; results from on-going trials with novel agents are awaited. 

As discussed, the R^3^i believes that monitoring of non-HDL cholesterol will provide a simple, practical tool for treatment decisions relating to lipid-related residual CV risk. However, evidence from the US National Health and Nutrition Examination Survey 2002–2010 shows that despite guidelines and the availability and use of effective lipid-modifying therapy, there has been little discernible improvement in non-HDL cholesterol goal attainment among patients with atherogenic dyslipidaemia. Of those with triglycerides > 200 mg/dL (2.3 mmol/L) only 13% had a non-HDL cholesterol level <130 mg/dL (3.4 mmol/L). Furthermore, over 60% had a high to very high non-HDL cholesterol level (>160 mg/dL or 4.1 mmol/L) [111]. Education is clearly needed to improve the implementation of guideline-based dyslipidaemia management.

The R^3^i recognises that current evidence relating to the treatment of residual atherogenic dyslipidaemia is somewhat limited. Consistent with the IAS position statement [39], the R^3^i recommends addition of a fibrate, niacin (North and South America), omega-3 fatty acids or ezetimibe as options for combination with a statin to reduce non-HDL cholesterol.

The future holds promise. Several emerging treatments may offer potential, although benefit versus risk analysis, especially in the longer-term, requires further consideration. The R^3^i awaits the results of major ongoing trials with these novel agents which may help to define the optimal management of atherogenic dyslipidaemia to reduce the clinical and socioeconomic burden of residual CV risk.

## Abbreviations

ACC/AHA: American College of Cardiology/American Heart Association; ACS: Acute coronary syndrome; Apo: Apolipoprotein; CETP: Cholesteryl ester transfer protein; CHD: Coronary heart disease; CI: Confidence interval; CV: Cardiovascular; CVD: Cardiovascular disease; EAS: European Atherosclerosis Society; EPA: Eicosapentaenoic acid; ER: Extended-release; HDL: High-density lipoprotein; IAS: International Atherosclerosis Society; LDL: Low-density lipoprotein; MI: Myocardial infarction; PCSK9: Proprotein convertase subtilisin/kexin type 9; PPAR: Peroxisome proliferator-activated receptor; R3i: Residual Risk Reduction Initiative; SPPARM: Selective peroxisome proliferator-activated receptor modulator; TRL: Triglyceride-rich lipoprotein; VLDL: Very low-density lipoprotein; ACCELERATE: A study of evacetrapib in high-risk vascular disease; ACCORD: Action to Control Cardiovascular Risk in Diabetes; AIM-HIGH: Atherothrombosis Intervention in Metabolic Syndrome with low HDL/high triglycerides: Impact on Global Health Outcomes; AleCardio: Aleglitazar in patients with a recent acute coronary syndrome and type 2 diabetes mellitus; ASSURE: ApoA-I Synthesis Stimulation and Intravascular Ultrasound for Coronary Atheroma Regression Evaluation; DYSIS: Dyslipidemia International Study; ENHANCE: Ezetimibe and Simvastatin in Hypercholesterolemia Enhances Atherosclerosis Regression; FIELD: Fenofibrate Intervention and Event Lowering in Diabetes; HPS2-THRIVE: Heart Protection Study 2-Treatment of HDL to Reduce the Incidence of Vascular Events; ILLUMINATE: Investigation of Lipid Level Management to Understand its Impact in Atherosclerotic Events; IMPROVE-IT: IMProved Reduction of Outcomes: Vytorin Efficacy International Trial; JELIS: Japan Eicosapentaenoic acid Lipid Intervention Study; ORIGIN: Outcome Reduction with an Initial Glargine Intervention; POSCH: Program on the Surgical Control of the Hyperlipidemia; PROCAM: Prospective Cardiovascular Münster Study; REVEAL: Randomized Evaluation of the Effects of Anacetrapib through Lipid-modification; 4S: Scandinavian Simvastatin Survival Study; SEAS: Simvastatin and Ezetimibe in Aortic Stenosis; SHARP: Study of Heart and Renal Protection.

## Competing interests

P Amarenco (PA) has received research grants from Pfizer, Sanofi, Bristol-Myers-Squibb, Merck, AstraZeneca, Boehringer Ingelheim and the French government; and honoraria for lectures/consultancy from Pfizer, Sanofi, Bristol-Myers-Squibb, Merck, AstraZeneca, Boehringer Ingelheim, Bayer, Daiichi Sankyo, Lundbeck, Edwards, Boston Scientific, Kowa, and St-Jude Medical.

P Barter (PB) has received research grants from Merck and Pfizer; honoraria for consulting from Amgen, AstraZeneca, ISIS, Kowa, Merck, Novartis, Pfizer and Roche; and honoraria as a member of Advisory Boards from AstraZeneca, CSL, Kowa, Lilly, Merck, Novartis, Pfizer and Roche.

J Betteridge (JB) has received honoraria for advisory boards and lectures from MSD, Pfizer, AstraZeneca, Kowa, Janssen, Amgen, Takeda and Sanofi.

E Bruckert (EB) has received research funding from GlaxoSmithKline, MSD, Genzyme, Sanofi, Aegerion and Montreal University; and honoraria for consulting/presentation from AstraZeneca, Genfit, Genzyme, MSD, Pfizer, Sanofi, Servier, AMT, Merck, Lilly, Novo-Nordisk, Pfizer and Aegerion.

A Cuevas (AC) has served on advisory boards for MSD and Amgen, and has received honoraria for lectures from MSD and Sanofi.

J Davignon (JD) has received honoraria for consultancy or as a scientific advisor for Abbott (Solvay), Acasti Pharma, Amgen, AstraZeneca, Anthera, Genzyme, McCain, Merck, Pfizer, Pharmena (Cortria), Sanofi-Regeneron, Roche and Valeant; and for participation in clinical trials for Amgen, Cortria, Genzyme, Merck, Pfizer and Sanofi-Regeneron. He has also received honoraria as a member of the Speakers bureau for the International Atherosclerosis Society. He is a Board Member for the Consortium Québecois sur la Découverte du Médicament (CQDM), and the Residual Risk Reduction Initiative Foundation.

E Ferrannini (EF) has received honoraria for speakers bureau/advisory boards from MSD, Halozyme, GlaxoSmithKline, Bristol-Myers-Squibb, AstraZeneca, Eli Lilly & Co., Novartis, Daiichi Sankyo and Sanofi. He has received research grant support from Eli Lilly & Co., and Boehringer Ingelheim.

M Farnier (MF) has received grants, consulting fees and/or honoraria for lectures for Abbott, Amgen, Boehringer Ingelheim, Genzyme, Kowa, Merck and Co., Novartis, Pfizer, Recordati, Roche, Sanofi-Aventis and Bristol-Myers-Squibb.

P Fioretto (PF) has received honoraria for lectures from Abbott, Bristol-Myers-Squibb, AstraZeneca, Boehringer and Lilly.

J-C Fruchart (JCF) has received honoraria as a consultant for SMB laboratories, McCain and Kowa Co. Ltd. He is President of the Residual Risk Reduction Initiative.

J Genest (JG) has received research funding from Amgen, Lilly and Merck and honoraria as a member of Speaker’s bureau/advisory boards from Merck, Amgen, Sanofi and Aegerion.

HN Ginsberg (HNG) has received research funds from Sanofi-Regeneron, Amgen, Sanofi-Genzyme, Merck and consulting honoraria from Sanofi-Regeneron, Amgen, Sanofi-Genzyme, Merck, Bristol-Myers-Squibb, AstraZeneca, Pfizer, Kowa, Janssen and Boehringer Ingelheim. 

AM Gotto (AMG) is on the board of Directors for Aegerion and Arisaph; has been a consultant for AstraZeneca, Janssen, Kowa, Merck, Pfizer and Roche; and has served on advisory boards for DuPont and Vatera Capital.

MP Hermans (MPH) has served on an advisory panel and/or received speaker’s honoraria or travel/research grants from Abbott, Amgen, AstraZeneca, Boehringer, Bristol-Myers-Squibb, Boehringer Ingelheim, GlaxoSmithKline, Janssen, Eli Lilly, LifeScan, Menarini, Merck, Novartis, Novo Nordisk, Roche, Sanofi and Takeda.

T Kodama (TK) has received honoraria as a consultant and research funding from Kowa Co.Ltd.

M Kremp (MK) has received honoraria for lectures from AstraZeneca, MSD, Bristol-Myers-Squibb and Sanofi.

J Millan Núñez-Cortés (JMN-C) has received honoraria as a member of advisory boards from Abbott, AstraZeneca, MSD, Pfizer and Sanofi; and for educational activities from Abbott, AstraZeneca and MSD.

H Ogawa (HO) has received honoraria for consulting from Amgen, GlaxoSmithKline and Novartis; and honoraria for lectures from AstraZeneca, Bayer, Boehringer lngelheim, Daiichi Sankyo, Mitsubishi Tanabe, MSD, Sanofi and Takeda. He has received research/scholarship grants from Bayer, Daiichi Sankyo, AstraZeneca, Astellas, Takeda, Mitsubishi Tanabe, Boehringer lngelheim and MSD.

J Plutzky (JP) has received research grants from GlaxoSmithKline and Bristol-Myers-Squibb; and honoraria for consultancy from Amylin Pharmaceuticals, AstraZeneca, Bristol-Myers-Squibb, Genzyme, GlaxoSmithKline, Eli Lilly, Janssen, Mesoblast, Merck, NovoNordisk, Pfizer, Roche/Genentech, Takeda and Vivus.

DJ Rader (DJR) has received honoraria for consulting from Merck, Pfizer, Eli Lilly, Sanofi, Amgen, Novartis, Omthera, Aegerion and CSL.

RD Santos (RDS) has received honoraria for consulting and/or speaking from AstraZeneca, Abbott, Biolab, Merck, Bristol-Myers-Squibb, Roche, Pfizer, Amgen, Aegerion, Boehringer Ingelheim, Sanofi, Genzyme and Nestle.

A Tall (AT) has received honoraria for lectures and advisory boards from MSD, Eli Lilly, Roche, Amgen, Arisaph and CSL.

L Tokgözoğlu (LT) has received honoraria for lectures and advisory boards from Abbott, Actelion, AstraZeneca, Bayer, Boehringer Ingelheim, Daiichi Sankyo, Kowa, MSD, Novartis, Pfizer, Roche, Sanofi and Servier.

PP Toth (PPT) has received honoraria as a member of speakers bureau for Amarin, AstraZeneca, GlaxoSmithKline, Kowa, Merck; and for consultancy for Amgen, AstraZeneca, Atherotech, Boehringer Ingelheim, Kowa, Liposcience and Merck.

P Valensi (PV) has given lectures and/or been a consultant for Abbott, MSD and Kowa.

C Wanner (CW) has received honoraria for lectures and travel support from Astellas, Merck and Pfizer.

A Zambon (AZ) has received speaker honoraria from Abbott, AstraZeneca, Roche and Amgen.

P Zimmet (PZ) has received travel funding from Fournier.

K Al-Rubeaan (KA-R), G Assmann (GA), Y Matsuzawa (YM), C Calvo Monfil (CCM), D Hu (DH),T Kadowaki (TK), S Sadikot (SS), E Shlyakhto (ES), P Sritara (PS), R Sy (RS), CE Tan (CET) and J Zhu (JZ) report no competing interests.

## Authors’ contributions

JCF, JD and MPH prepared the initial draft of the manuscript; GA also provided data relating to the PROCAM study. All authors critically reviewed the paper, were involved in revisions of the manuscript, and read and approved the final manuscript.

## Authors’ information

The Residual Risk Reduction Initiative (http://www.r3i.org) is an international, academic, multidisciplinary non-profit organization which is focused on addressing the high residual risk of macrovascular and microvascular complications in patients with atherogenic dyslipidaemia.

G Assmann is also chairman of the Assmann Foundation for Prevention.
